# Synergism Between Bacterial GAPDH and OMVs: Disparate Mechanisms but Co-Operative Action

**DOI:** 10.3389/fmicb.2015.01231

**Published:** 2015-11-09

**Authors:** David E. Whitworth, Bethan H. Morgan

**Affiliations:** Institute of Biological, Environmental and Rural Sciences, Aberystwyth UniversityAberystwyth, UK

**Keywords:** fusogen, extracellular vesicles, *Myxococcus xanthus*, secretion, virulence, pathogenesis

## Introduction

Outer membrane vesicles (OMVs) shed from bacteria contribute to pathogenesis by promoting colonization of host tissues and trafficking virulence factors into host cells via fusion with the host cell plasma membrane. Glyeraldehyde-3-phosphate dehydrogenase (GAPDH) is also secreted by prokaryotes, but enhances pathogenesis by promoting adhesion of bacteria to host cell surfaces. However, GAPDH is also known to catalyze the fusion of membranes, and it has been shown to promote OMV activity in the non-pathogen *Myxococcus xanthus*. We suggest that during infection by Gram-negative bacteria, GAPDH and OMVs work synergistically to stimulate pathogenesis.

## Outer membrane vesicles in health and disease

**Figure 1:**
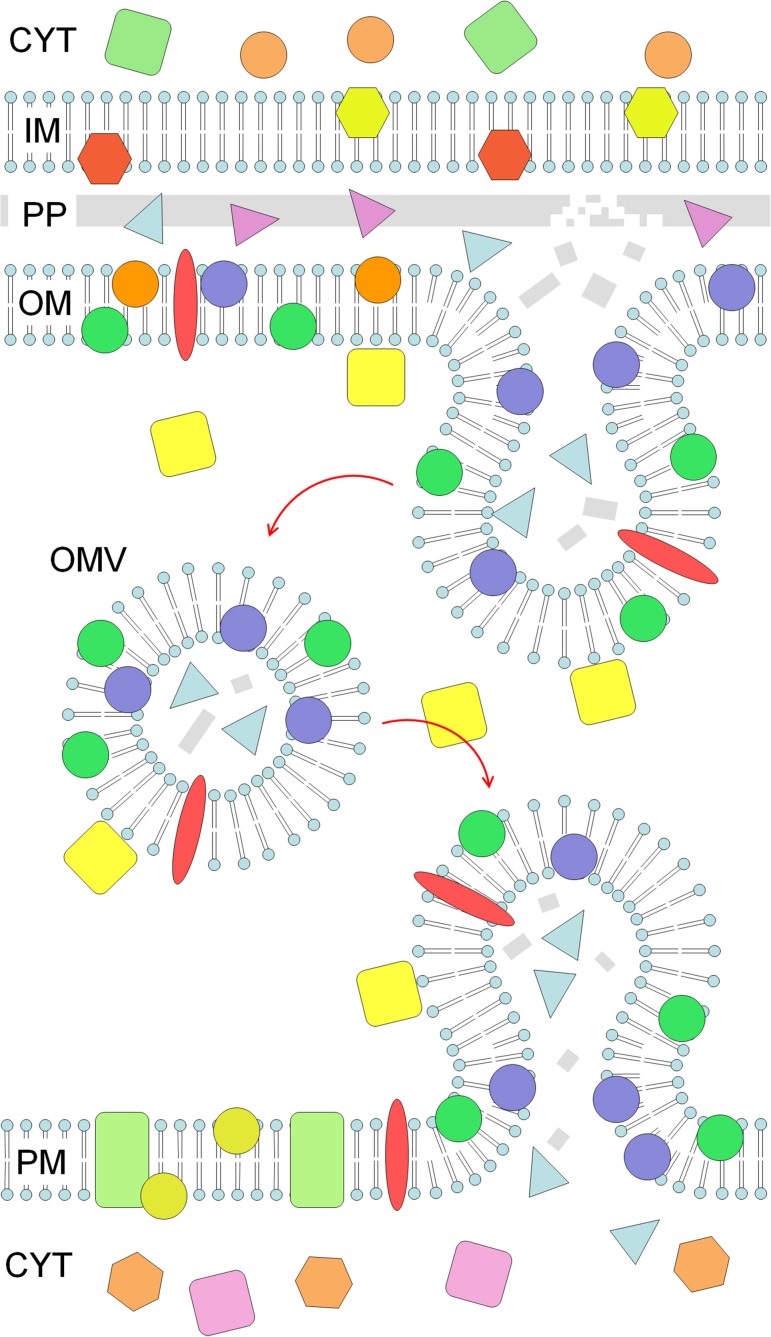
**OMV production and targeting to a eukaryotic cell**. A Gram-negative cell (**top**) produces an OMV (**middle**) by pinching-off a protrusion of the outer membrane (OM). The OMV is enriched in a subset of OM and periplasmic (PP) material, including specific proteins and peptidoglycan fragments (gray), while inner membrane (IM) and cytoplasmic (CYT) material is absent. The OMV is able to fuse with a target membrane (**bottom**), in this case the plasma membrane (PM) of a eukaryotic cell, delivering its contents into the PM and cytoplasm (CYT). GAPDH (yellow squares) is found on the surface of cells and OMVs, and can stimulate the fusion of OMVs with target membranes.

A common bacterial mechanism for engineering the environment involves the secretion of OMVs—10–300 nm diameter packages, pinched off from the outer membrane of Gram-negative bacteria, enclosing periplasmic material (Figure 1). OMV constituents can be specifically targeted for inclusion in OMVs, however the mechanisms of OMV biogenesis and cargo targeting remain poorly defined (Kulkarni and Jagannadham, 2014).

OMVs are able to migrate away from their producing cells, accessing niches unavailable to the producing cell, and delivering secreted material to distant sites of action. Packaging within OMVs means their contents are not diluted as they are transported far from the cell, are protected from the environment (e.g., extracellular proteases), and cargo complexes can be secreted as pre-assembled entities (Ellis and Kuehn, 2010; Kulkarni and Jagannadham, 2014). At their site of action, OMVs can deliver their contents by two mechanisms. They can fuse with target membranes (Figure 1; Kadurugamuwa and Beveridge, 1999; Bomberger et al., 2009), or contact with a surface can trigger OMV lysis (Kadurugamuwa and Beveridge, 1996), releasing OMV contents.

OMVs are produced by all Gram-negative bacteria, and are known to have diverse antimicrobial, biofilm-promoting, virus-resistance, quorum-signaling and virulence-enhancing properties (Manning and Kuehn, 2013). The virulence of pathogens is known to correlate with the degree of vesiculation (Rolhion et al., 2005), and OMVs are able to enhance colonization of host tissues, modify host cell biology, and/or protect the OMV-producer from therapeutics and the host immune response (Inagaki et al., 2006; Thay et al., 2014; Vanhove et al., 2015).

OMV production is induced by stresses associated with host colonization (McBroom and Kuehn, 2007), for example by exposure to host muscle tissue (Dutson et al., 1971). They are able to adhere to host cells (Inagaki et al., 2006), and promote biofilm formation in clinically important bacteria (Grenier and Mayrand, 1987; Kamaguchi et al., 2003; Yonezawa et al., 2009). The OMVs of many pathogens have been documented to contain toxins and other virulence factors (Elluri et al., 2014; Roier et al., 2014; Thay et al., 2014; Vanhove et al., 2015), and OMV-packaging has been shown to stabilize, activate and/or regulate toxin activity (Fahie et al., 2013; Bielaszewska et al., 2014; Elluri et al., 2014).

## The gifted enzyme glyceraldehyde-3-phosphate dehydrogenase

GAPDH (EC 1.2.1.12) is first encountered by biology students as an essential enzyme of central metabolism. It is a highly conserved protein, typically found as a tetramer (Seidler, 2013), and can be post-translationally modified in multiple ways (Sirover, 2014).

Intriguingly, GAPDH has been ascribed many additional roles beyond metabolism in eukaryotes, including glycosylation of uracil in DNA, transcriptional activation and apoptotic regulation (Sirover, 2005). One of its more exotic “moonlighting” activities is the ability to fuse membranes together (Glaser and Gross, 1995). This can occur *in vitro*, but has also been implicated in the fusion of secretory granules with the plasma membrane in neutrophils, fusion of presynaptic vesicles with the synaptic membrane (and their loading with cargo), axoplasmic transport, ER-Golgi vesicular shuttling, and nuclear membrane fusion (Glaser and Gross, 1995; Hessler et al., 1998; Ikemoto et al., 2003; Nakagawa et al., 2003). The structural basis of fusogenesis is unknown, however fusion requires binding to the relatively scarce membrane lipid phosphatidylserine (PS), and the PS binding site of GAPDH has been elucidated (Kaneda et al., 1997).

The classic glycolytic role of GAPDH places it in the cytoplasm, and it lacks an N-terminal signal sequence or other trafficking motif. However, with the advent of proteomics, many studies have identified GAPDH in extracellular fractions of a wide range of bacteria (Curtis et al., 2007; Holland et al., 2010; Deng et al., 2012; Vanden Bergh et al., 2013; Wang et al., 2013). It is a major surface protein of Gram-positive (Pancholi and Fischetti, 1992; Pasztor et al., 2010; Oliveira et al., 2012), and Gram-negative bacteria (Egea et al., 2007; Gao et al., 2014). In streptococci its release beyond the cell involves autolysis, with released protein then specifically binding to the surface of unlysed cells (Terrasse et al., 2015). Thus, GAPDH seems to be an almost ubiquitous protein, being commonly found within cells, on cells and beyond cells.

Extracellular bacterial GAPDH promotes adhesion to and invasion of host tissue, inhibits host lysozyme, and triggers apoptosis in macrophages (Seidler and Seidler, 2013). During host colonization, it is known to adhere to a variety of substrates, including PS, mucin, plasminogen and fibrinogen (Alvarez et al., 2003; Egea et al., 2007; Gao et al., 2014). It is likely that further mechanisms exist by which GAPDH promotes virulence, but studies have been hampered by difficulties in deleting the gene encoding GAPDH, due to its essential role in energy metabolism (Henderson and Martin, 2011).

## OMVs and GAPDH working together

The soil-dwelling myxobacterium *M. xanthus* is a predator of a wide range of bacteria and fungi, and OMVs are implicated in several aspects of its life-cycle (Whitworth, 2011). Its OMVs are loaded with hydrolases and they are able to kill other microbes, including *Escherichia coli* and *Pseudomonas aeruginosa* (Evans et al., 2012). Adding GAPDH to *M. xanthus* OMVs enhances their ability to kill prey cells. This is attributable to the fusogenic activity of the enzyme, as only intact OMVs exhibit cytotoxic activity (Evans et al., 2012), and OMVs of other bacteria are known to kill prey cells through fusion with their outer membrane (Kadurugamuwa and Beveridge, 1996). GAPDH has been found to be a major component of *M. xanthus* cells, OMVs and soluble secretome (Whitworth et al., 2015) suggesting GAPDH stimulates the antimicrobial activity of *M. xanthus* OMVs in the wild, by promoting their fusion with prey cells.

EVpedia, the EV database (Kim et al., 2013), shows that GAPDH has been observed as a component of the OMVs of many organisms (including *E. coli, P. aeruginosa, Edwardsiella tarda, Francisella tularensis, Francisella philomiragia, Acinetobacter baumanii*, and *Neiserria meningiditis*). Given the virtual ubiquity of GAPDH and OMV secretion, it is possible they could be working together in other contexts. The bacterial behavior for which there is most evidence of potential GAPDH-OMV synergy is pathogenesis. If the activities analogous to those observed for *M. xanthus* occur for pathogens *in vivo*, then pathogen OMVs would be stimulated to fuse with target cells/membranes by pathogen-derived GAPDH.

## GAPDH/OMV co-operation during pathogenesis

There are several lines of evidence described above which suggest such GAPDH/OMV synergy:

- Both GAPDH and OMVs are secreted commonly (ubiquitously?) by pathogens.
- Both GAPDH and OMVs stimulate pathogenesis.
- GAPDH is a common component of OMVs.
- GAPDH is an adhesin, but also has membrane fusion activity.
- OMVs can deliver their contents beyond target membranes by fusing with them.
- GAPDH can enhance OMV activity by stimulating membrane fusion.

Pathogen-derived GAPDH has been shown to have a mechanistic role in tissue colonization and adherence, but in no other aspects of the pathogenicity of Gram-negative organisms. However, making a topological mutant that does not secrete GAPDH results in a strain with reduced (but not abolished) host cell adherence (Boël et al., 2005), indicating that pathogens have other adhesins that complement GAPDH's matrix-binding activity. Nevertheless, non-pathogenic strains of *E. coli* do not secrete GAPDH (Egea et al., 2007), which is taken as evidence that GAPDH is required for pathogenicity. Together these observations suggest that GAPDH has a role in virulence beyond just adhesion.

The few studies that have demonstrated membrane fusion by OMVs have taken no effort to reduce GAPDH levels/activity in their OMV preparations, and the organisms whose OMVs are known to fuse with membranes are also known to naturally contain GAPDH. GAPDH may be merely promoting an intrinsic OMV activity, but the possibly cannot be discounted that GAPDH is actually required for OMV membrane-fusion activity and resulting toxin delivery.

An interesting mechanistic feature common to OMV uptake and GAPDH-catalyzed membrane fusion is that both processes are thought to be dependent on specific lipids. The fusogenic activity of GAPDH requires cholesterol and the ether lipid plasmenylethanolamine, which are both commonly found in mammalian membranes (Glaser and Gross, 1995). Kesty et al. (2004) showed that enterotoxigenic *E. coli* secretes enterotoxin via OMVs, and that host cells were able to endocytose the toxin-containing OMVs by a mechanism dependent on cholesterol-rich lipid rafts. In principle, GAPDH could stimulate OMVs to bind to cholesterol-rich membranes, which are then prime substrates for GAPDH-mediated fusion or host-mediated endocytosis (with delivery of OMV contents into the target cell).

There is also the potential for OMVs to affect GAPDH function reciprocally. OMVs increase the effective amount of bacterial OM, which GAPDH can cross-link by virtue of its properties as an adhesin, potentially promoting biofilm formation and uptake/fusion of OMVs.

## Beyond pathogenesis

As OMVs and GAPDH appear to be ubiquitously secreted by Gram-negative bacteria, it is likely that GAPDH will be implicated in other functions of OMVs. Biofilm formation is an important and universal phenomenon, promoted by OMVs. It is also promoted by intercellular quorum signaling which itself can be transduced through OMVs (Mashburn and Whiteley, 2005). Mixed biofilms are frequently observed in nature, and competition between the different inhabitants is important for determining fitness. Delivery of toxins to competitors or prey organisms via OMVs has been observed and thus modulation of OMV activity by GAPDH would likely be an important fitness determinant.

In the laboratory, several obvious experiments arise from considering the potential involvement of GAPDH in OMV activity.

- No bacterial GAPDH has yet been shown to possess fusogenic activity and this needs to be confirmed, perhaps by monitoring lipid/content mixing through fluoresence quenching/enhancement (Glaser and Gross, 1994). Care would need to be taken however as GAPDH-mediated membrane fusion may be dependent on membrane lipid composition as it is in eukaryotes (Glaser and Gross, 1995).
- We would expect GAPDH-depleted OMVs to be impeded in their ability to fuse with target membranes. This would be a technically challenging prediction to test however, due to the important metabolic role of GAPDH precluding facile gene deletion, and the inherent membrane-binding affinity of GAPDH defying physical removal. Nevertheless, it should be possible to engineer a GAPDH deletant by developing appropriate media to support metabolic bypassing of glycolysis/gluconeogenesis in the mutant. Alternatively a “functional” mutant could be created by placing the GAPDH gene under the control of an inducible or repressible promoter, or through the creation of a topologically restricted version of GAPDH (Boël et al., 2005). GAPDH inhibitors are also available, which might also affect fusogenic activity [e.g., pentalenolactone and koningic (heptelidic) acid].
- GAPDH is expected to promote adhesion between bacterial cells, as well as cell-OMV adhesion. It would be interesting to see whether reducing membrane-associated GAPDH levels does impact negatively on colonial growth and/or aggregation. Perhaps by using simple assays which monitor sedimentation of aggregated cells, for example the approach used by Chang and Dworkin (1994) to measure bacterial cohesion.
- The effect of cholesterol and/or ether lipids on GAPDH-mediated OMV-membrane fusion should be tested for a range of OMV producers and target membranes, to delineate any conservation of lipid requirements.

## Implications

Bacterial GAPDH has already proven useful as a therapeutic target with the development of cross-protective GAPDH-based vaccines against Gram-negative and -positive bacteria for agri- and aqua-culture (Li et al., 2011; Vanden Bergh et al., 2013; Velineni and Timoney, 2013; Trung Cao et al., 2014). The GAPDH inhibitor pentalenolactone (Cane and Sohng, 1994) is known to act as an antibiotic due to its disruption of bacterial glycolysis, but it is also potent against mammalian homologs and is not used in the clinic. Nevertheless, there are enough sequence differences between human and bacterial GAPDH to make GAPDH-targeted therapies for the clinic plausible (Seidler and Seidler, 2013), and such inhibitors could also be useful beyond the clinic as antibiofilm/antifouling compounds.

OMVs are proving efficacious as hapten components of antibacterial vaccines (Acevedo et al., 2014; Choi et al., 2014; Nieves et al., 2014), and as adjuvants for delivery of heterologous haptens (Moshiri et al., 2012). Perhaps part of the success of OMV vaccines is because they are multivalent GAPDH-presenting entities. Rationally combining GAPDH and OMVs within vaccines has the potential to synergistically enhance immunogenicity of each component. It is plausible that OMVs could also see use in the clinic as antimicrobials. Not only have they been shown to kill bacteria directly but they can also act as delivery devices for antibiotics (Kadurugamuwa and Beveridge, 1998). Potentially, the addition of stimulatory “accessory proteins” such as GAPDH would help make such OMV-based approaches more effective.

Beyond the clinic, a holistic understanding of the interaction between GAPDH and OMVs will need to consider the relative physical location of both entities and modulators of their activities. This will be especially important when considering mixed communities of bacteria, expressing a range of OMVs and GAPDH isoforms with differing target specificities and fusogenic potential. However, an enhanced understanding of such processes will provide invaluable information regarding the mechanisms of bacterial competition and co-operation.

## Author contributions

DW and BM conceived, drafted, and edited the work.

### Conflict of interest statement

The authors declare that the research was conducted in the absence of any commercial or financial relationships that could be construed as a potential conflict of interest.
